# Factors associated with optimal pharmacy refill adherence for antiretroviral medications and plasma HIV RNA non-detectability among HIV-positive crack cocaine users: a prospective cohort study

**DOI:** 10.1186/s12879-016-1749-y

**Published:** 2016-08-27

**Authors:** Kanna Hayashi, Evan Wood, Thomas Kerr, Huiru Dong, Paul Nguyen, Cathy M. Puskas, Silvia Guillemi, Julio S. G. Montaner, Michael-John Milloy

**Affiliations:** 1 British Columbia Centre for Excellence in HIV/AIDS, St. Paul’s Hospital, 608 - 1081 Burrard Street, Vancouver, BC V6Z 1Y6 Canada; 2 Division of AIDS, Department of Medicine, University of British Columbia, 608 - 1081 Burrard Street, Vancouver, BC V6Z 1Y6 Canada; 3 Faculty of Health Sciences, Simon Fraser University, 8888 University Drive, Burnaby, BC V5A 1S6 Canada

**Keywords:** Crack cocaine, ART adherence, Plasma HIV-1 RNA viral load suppression, Addiction treatment

## Abstract

**Background:**

Crack cocaine use is known to contribute to poor adherence to antiretroviral medications; however, little is known about facilitators of or barriers to effective HIV treatment use among HIV-infected crack cocaine users. We sought to identify correlates of optimal pharmacy refill adherence for antiretroviral medications and plasma HIV RNA viral load (pVL) suppression among this population.

**Methods:**

Data from a prospective cohort of HIV-positive people who use illicit drugs in Vancouver, Canada, were linked to comprehensive HIV clinical monitoring and pharmacy dispensation records. We used multivariable generalized linear mixed-effects modelling to longitudinally identify factors associated with ≥95 % adherence to pharmacy refills for antiretroviral medications and pVL <50 copies/mL among crack cocaine users exposed to highly-active antiretroviral therapy (HAART).

**Results:**

Among 438 HAART-exposed crack cocaine users between 2005 and 2013, 240 (54.8 %) had ≥95 % pharmacy refill adherence in the previous 6 months at baseline. In multivariable analyses, homelessness (adjusted odds ratio [AOR]: 0.58), ≥daily crack cocaine smoking (AOR: 0.64), and ≥ daily heroin use (AOR: 0.43) were independently associated with optimal pharmacy refill adherence (all *p* < 0.05). The results for pVL non-detectability were consistent with those of medication adherence, except that longer history of HAART (AOR: 1.06), receiving a single tablet-per-day regimen (AOR: 3.02) and participation in opioid substitution therapies was independently associated with pVL non-detectability (AOR: 1.55) (all *p* < 0.05).

**Conclusions:**

Homelessness, and daily crack cocaine and/or heroin use were independently and negatively associated with optimal HAART-related outcomes. With the exception of opioid substitution therapies, no addiction treatment modalities assessed appeared to facilitate medication adherence or viral suppression. Evidence-based treatment options for crack cocaine use that also confer benefits to HAART need to be developed.

## Background

Highly-active antiretroviral therapy (HAART) has substantially improved the life expectancy of people living with HIV [1] and has also been shown to prevent HIV transmission by reducing individuals’ viral load to undetectable levels [2, 3]. Despite the remarkable impact of HAART on the HIV/AIDS pandemic, its benefits on disease progression and the incidence of new infections have yet to be fully realized among all HIV-seropositive groups, such as people who use illicit drugs. Crack cocaine use has been shown to be an independent risk factor of HIV seroconversion in some settings, presumably through high-risk sexual activities associated with crack use [4–7]. While evidence-based strategies to reduce HIV transmission risk through injection drug use exist, such as needle and syringe programs [8], crack cocaine is often smoked, and interventions to reduce sexual risk behaviour among crack cocaine smokers have proven to be of limited effectiveness [5, 9, 10].

In 2014, the Treatment-as-Prevention (TasP) approach, which aims to expand access and adherence to HAART among people living with HIV and thereby prevent HIV transmission, was incorporated into the global HIV/AIDS response strategies by the Joint United Nations Programme on HIV/AIDS [11]. This is likely a potentially effective HIV prevention approach to address HIV transmission associated with persistent high-risk behaviour among HIV-infected crack cocaine users [12]. However, there remains concern regarding the viability of the intervention due to previous studies describing sub-optimal adherence to HAART in crack cocaine using populations [13–15]. For example, a 2002 study of HIV-infected people who use drugs reported that the strongest predictor of poor adherence to antiretroviral medications was cocaine/crack cocaine use [13]. A more recent study found that stimulant users, particularly those who used cocaine/crack cocaine, had seven times higher odds of HAART adherence failure (i.e., <90 % of doses taken) compared to patients who did not use drugs [15].

Despite many reports of poor adherence to HAART among HIV-infected crack cocaine users [13–15], to date, there is a paucity of research identifying factors associated with HAART adherence and plasma HIV RNA viral load (pVL) suppression in this population, which may be amenable to intervention. This is a concern as the success of TasP-based approaches relies on ensuring access and adherence to HAART. In this study, we sought to identify correlates of optimal adherence to antiretroviral medications and pVL non-detectability among HAART-exposed crack cocaine users in Vancouver, Canada, where crack cocaine use is common and has been identified as an independent predictor of HIV seroconversion [7].

## Methods

### Study design

Data for this analysis were derived from the AIDS Care Cohort to evaluate Exposure to Survival Services (ACCESS), a prospective cohort study of HIV-positive people who use illicit drugs in Vancouver, Canada. Recruitment and data collection procedures have been described in detail elsewhere [16]. In brief, beginning in 1996, participants were recruited through self-referral and street-based outreach from Vancouver’s Downtown Eastside neighbourhood, a post-industrial area with a large open drug market and high levels of illicit drug use, poverty, and HIV infection. Individuals were eligible to participate in ACCESS if they were aged 18 years or older, were HIV-seropositive, had used illicit drugs other than cannabis in the month prior to enrolment, and provided written informed consent. Participants were compensated $20 CAD at each study visit.

At baseline and semi-annually thereafter, participants completed an interviewer-administered questionnaire soliciting demographic data, information on drug use patterns, as well as other characteristics and exposures. At each visit, individuals also underwent an examination by a study nurse and provided blood samples for serologic analyses. Through a confidential linkage with the provincial Drug Treatment Program (DTP), a complete clinical profile of all CD4 T-cell counts, pVL observations, exposure to specific antiretroviral agents, and treatment outcomes for each participant were obtained. In British Columbia, all provision of HIV treatment is centralized through a province-wide dispensation program, where treatment and related care are provided free of charge. Data collection by the DTP has been described previously [17, 18]. The ACCESS study has been approved by the University of British Columbia/Providence Health Care Research Ethics Board.

### Participants and measures

Participants were eligible for the present analysis if they were recruited by ACCESS between December 1, 2005 and November 30, 2013, reported having smoked crack cocaine in the six months prior to the baseline assessment, and had initiated HAART prior to enrolment in ACCESS. As well, at least one observation of CD4 cell count and pVL had to be completed within ±180 days of the day they entered the study. For a sub-analysis focusing on dual crack cocaine and opioid users, the sample was further restricted to those who had a history of any opioid use or participation in methadone maintenance therapy at baseline.

The primary outcomes of interest were optimal adherence to pharmacy refills for antiretroviral medications and pVL non-detectability in the previous six months. We measured pharmacy refill adherence in each 6-month period as the number of days for which medications were dispensed over the number of days since the initiation of HAART, which was capped at 180 days. We defined optimal pharmacy refill adherence as equal to or greater than 95 %. We have previously demonstrated the clinical validity of this pharmacy refill data and shown that it reliably predicts virologic suppression [19, 20] and survival [21]. Plasma HIV RNA viral load non-detectability was defined as having achieved a HIV RNA viral load of <50 copies/mL of plasma. In the event that more than one pVL observation was made within a 6-month follow-up period, we used the median of all the observations, which was then categorized into either <50 or ≥50 copies/mL of plasma.

We considered a range of explanatory variables that might be associated with optimal pharmacy refill adherence and/or pVL non-detectability. Demographic characteristics included: age (per 10-year older), gender (male *vs.* female), ethnicity (Caucasian *vs.* other), and education level (<secondary education *vs.* ≥secondary education). Substance-using behaviours included: crack cocaine smoking (≥daily *vs.* <daily), heroin use (≥daily *vs.* <daily), crystal methamphetamine use (≥daily *vs.* <daily), and heavy alcohol use (≥5 drinks per day *vs.* <5 drinks per day). Other behavioural variables included: homelessness, engagement in sex work or drug dealing, incarceration, participation in inpatient addiction treatment (i.e., recovery houses, detoxification facilities, and treatment centres), participation in outpatient detoxification services, seeing drug counsellors or participation in addiction-related peer support meetings (e.g., Narcotics Anonymous, Cocaine Anonymous, etc.), and participation in opioid substitution therapies (i.e., methadone maintenance therapy or Suboxone treatment) (all: yes *vs.* no). The use of opioid substitution therapies was considered in the sub-analysis focusing on dual crack cocaine and opioid users only. Clinical variables included: CD4 cell count (per 100-cells/mm^3^ increase); antiretroviral tablet regimen (single tablet *vs.* 2-3 tablets *vs.* >3 tablets per day); years since HAART initiation (per year increase); physician experience on HIV care at the time of antiretroviral therapy initiation (having ever treated <6 patients *vs.* ≥6 patients). All variables except for the demographic characteristics referred to the previous six months unless otherwise indicated.

### Statistical analyses

First, we examined baseline characteristics of the sample, stratified by medication adherence (≥95 % *vs.* <95 %) in the six months prior to the baseline assessment. Categorical variables were analysed using Pearson’s χ^2^ test (or Fisher’s exact test in the presence of small sell counts) and continuous variables were analysed using the Wilcoxon rank-sum test. Next, we used generalized linear mixed-effects modelling (GLMM) with the logit link to longitudinally identify factors associated with optimal pharmacy refill adherence and pVL non-detectability, respectively. This form of regression modelling accounts for the correlation between repeated observations gathered over time from the same individual, and estimates the effect of the explanatory variables on the likelihood of optimal pharmacy refill adherence and pVL non-detectability in each individual.

Since our study aimed to identify the set of variables that best explains a higher odds of optimal pharmacy refill adherence and pVL non-detectability, respectively, we used *a priori*-defined statistical protocol based on the examination of the Akaike information criterion (AIC) and Type III *p*-values to construct multivariable GLMM logistic regression models. This procedure balances model selection on finding the best explanatory model with best model fit, as described previously [22]. In brief, we first included in the full multivariable models all explanatory variables that were significantly associated with optimal pharmacy refill adherence and pVL non-detectability, respectively, at the *p* < 0.10 level in the univariable analyses. After examining the AIC value of the models, we removed the variable with the largest *p*-value and built a reduced model. We continued this iterative process and selected the multivariable models with the lowest AIC value. Through this process, some variables, even though being significantly associated with an outcome at the level of *p* < 0.05 in bivariable analyses, were dropped from the final multivariable model. For the sub-analysis focusing on dual crack cocaine and opioid users, we added participation in opioid substitution therapies to the final multivariable models as a covariate. All *p*-values were two-sided. All statistical analyses were performed using the SAS software version 9.3 (SAS, Cary, NC).

## Results

In total, 438 HAART-exposed crack cocaine users were included in this study, of whom 293 (66.9 %) were male and 239 (54.6 %) self-reported Caucasian ancestry. Of the 438 participants, 406 (92.7 %) had returned for at least one follow-up visit, contributing a median follow-up duration of 48.0 months (interquartile range [IQR]: 35.0 – 75.9). At baseline, 240 (54.8 %) exhibited optimal pharmacy refill adherence in the previous six months, and a total of 390 (89.0 %) individuals attained optimal adherence at some point during the study period.

Table 1 presents sample characteristics at baseline, stratified by medication adherence levels. A substantial proportion of the sample was homeless (29.5 %), smoked crack cocaine at least on a daily basis (40.0 %), and engaged in sex work or drug dealing (37.4 %) in the previous six months. Very few individuals accessed any types of addiction treatment except for opioid substitution therapies (1.1–9.8 %), while the majority (63.5 %) of 293 dual crack cocaine and opioid users participated in opioid substitution therapies in the previous six months.

**Table 1:** Baseline characteristics of HAART-exposed crack cocaine users in Vancouver, Canada, stratified by optimal pharmacy refill adherence (*n* = 438)

Characteristic	Total (*n* = 438) *n* (%)	≥95 % pharmacy refill adherence for antiretrovirals^a^		*p*-value
		Yes 240 (54.8 %)	No 198 (45.2 %)	
Age (median, IQR)	44 (38–49)	45 (40–51)	42 (37–46)	<0.001
Gender				
Male	293 (66.9 %)	171 (71.3 %)	122 (61.6 %)	0.033
Female	145 (33.1 %)	69 (28.7 %)	76 (38.4 %)	reference
Ancestry				
Caucasian	239 (54.6 %)	147 (61.3 %)	92 (46.5 %)	0.002
Non-Caucasian	199 (45.4 %)	93 (38.7 %)	106 (53.5 %)	reference
Education				
< Secondary education	228 (52.1 %)	116 (48.3 %)	112 (56.6 %)	0.069
≥ Secondary education	196 (44.7 %)	117 (48.8 %)	79 (39.9 %)	reference
Homeless^a^				
Yes	129 (29.5 %)	57 (23.8 %)	72 (36.4 %)	0.003
No	306 (69.9 %)	182 (75.8 %)	124 (62.6 %)	reference
Crack cocaine smoking^a^				
≥ Daily	175 (40.0 %)	78 (32.5 %)	97 (49.0 %)	0.001
< Daily	263 (60.0 %)	162 (67.5 %)	101 (51.0 %)	reference
Heroin use^a^				
≥ Daily	48 (11.0 %)	19 (7.9 %)	29 (14.7 %)	0.024
< Daily	389 (88.8 %)	221 (92.1 %)	168 (84.9 %)	reference
Crystal methamphetamine use^a,c^				
≥ Daily	9 (2.1 %)	5 (2.1 %)	4 (2.0 %)	1.000
< Daily	428 (97.7 %)	235 (97.9 %)	193 (97.5 %)	reference
Heavy alcohol use (≥5 drinks per day)^a,c^				
Yes	13 (3.0 %)	5 (2.1 %)	8 (4.0 %)	0.266
No	425 (97.0 %)	235 (97.9 %)	190 (96.0 %)	reference
Engagement in sex work or drug dealing^a^				
Yes	164 (37.4 %)	78 (32.5 %)	86 (43.4 %)	0.018
No	270 (61.6 %)	160 (66.7 %)	110 (55.6 %)	reference
Incarceration^a^				
Yes	58 (13.2 %)	32 (13.3 %)	26 (13.1 %)	0.951
No	380 (86.8 %)	208 (86.7 %)	172 (86.9 %)	reference
HIV physician experience^b^				
Previously treated <6 patients	48 (11.0 %)	25 (10.4 %)	23 (11.6 %)	0.397
Previously treated ≥6 patients	340 (77.6 %)	199 (82.9 %)	141 (71.2 %)	reference
CD4 cell count^a^ (median, IQR)	300 (180–450)	340 (223.5–495)	235 (120–360)	<0.001
Years since HAART initiation^a^ (median, IQR)	5.7 (2.2–8.5)	4.9 (1.6–8.0)	6.9 (3.2–8.8)	<0.001
Regimen^b^				
Single tablet per day	34 (7.8 %)	24 (10.0 %)	10 (5.1 %)	0.278
2-3 tablets per day	121 (27.6 %)	76 (31.7 %)	45 (22.7 %)	0.723
> 3 tablets per day	230 (52.5 %)	140 (58.3 %)	90 (45.5 %)	reference
Participation in inpatient addiction treatment^a^				
Yes	35 (8.0 %)	19 (7.9 %)	16 (8.1 %)	0.950
No	403 (92.0 %)	221 (92.1 %)	182 (91.9 %)	reference
Participation in outpatient detoxification or other outpatient addiction treatment^a,c^				
Yes	5 (1.1 %)	4 (1.7 %)	1 (0.5 %)	0.384
No	433 (98.9 %)	236 (98.3 %)	197 (99.5 %)	reference
Seeing drug counsellors or participation in addiction-related peer support meetings^a^				
Yes	43 (9.8 %)	31 (12.9 %)	12 (6.1 %)	0.016
No	395 (90.2 %)	209 (87.1 %)	186 (93.9 %)	reference
*Restricted to dual crack cocaine and opioid users (n = 293)*				
Participation in opioid substitution therapies^a^				
Yes	186 (63.5 %)	115 (70.6 %)	71 (54.6 %)	0.005
No	107 (36.5 %)	48 (29.4 %)	59 (45.4 %)	reference

*HAART* highly active antiretroviral therapy, *CI* confidence interval, *IQR* interquartile range

^a^ denotes activities/events in the past 6 months

^b^ denotes at the time of antiretroviral therapy initiation

^c^ p-values were derived from Fisher’s exact test

Not all cells added up to 100 % due to some missing values

Compared to those who had suboptimal pharmacy refill adherence in the previous six months, individuals who had optimal pharmacy refill adherence were older, more likely to be male, self-report Caucasian ancestry, possess a high school diploma, have higher CD4 cell count and a shorter length of exposure to HAART, and have seen drug counsellors or participated in addiction-related peer support groups in the previous six months, while they were less likely to be homeless, smoke crack cocaine or use heroin at least daily, engage in sex work or drug dealing in the previous six months (all *p* < 0.05). Additionally, among a sub-sample of dual crack cocaine and opioid users, those who accessed opioid substitution therapies were more likely to have exhibited optimal pharmacy refill adherence (*p* = 0.005). Table 2 presents baseline characteristics stratified by pVL non-detectability. The observed significant associations were generally similar to those found with optimal pharmacy refill adherence, except that those who were on a 2-3 tablet-per-day regimen were more likely to have pVL non-detectability compared to those who were on a >3 tablet-per-day regimen (*p* = 0.008).

**Table 2:** Baseline characteristics of HAART-exposed crack cocaine users in Vancouver, Canada, stratified by pVL non-detectability (*n* = 438)

Characteristic	Total (*n* = 438) *n* (%)	pVL <50 copies/mL^a^		*p*-value
		Yes 212 (48.4 %)	No 226 (51.6 %)	
Age (median, IQR)	44 (38–49)	45 (41–51)	42 (36–47)	<0.001
Gender				
Male	293 (66.9 %)	149 (70.3 %)	144 (63.7 %)	0.145
Female	145 (33.1 %)	63 (29.7 %)	82 (36.3 %)	reference
Ancestry				
Caucasian	239 (54.6 %)	122 (57.5 %)	117 (51.8 %)	0.225
Non-Caucasian	199 (45.4 %)	90 (42.5 %)	109 (48.2 %)	reference
Education				
< Secondary education	228 (52.1 %)	107 (50.5 %)	121 (53.5 %)	0.462
≥ Secondary education	196 (44.7 %)	99 (46.7 %)	97 (42.9 %)	reference
Homeless^a^				
Yes	129 (29.5 %)	36 (17.0 %)	93 (41.2 %)	<0.001
No	306 (69.9 %)	174 (82.1 %)	132 (58.4 %)	reference
Crack cocaine smoking^a^				
≥ Daily	175 (40.0 %)	72 (34.0 %)	103 (45.6 %)	0.013
< Daily	263 (60.0 %)	140 (66.0 %)	123 (54.4 %)	reference
Heroin use^a^				
≥ Daily	48 (11.0 %)	16 (7.5 %)	32 (14.2 %)	0.026
< Daily	389 (88.8 %)	196 (92.5 %)	193 (85.4 %)	reference
Crystal methamphetamine use^a,c^				
≥ Daily	9 (2.1 %)	6 (2.8 %)	3 (1.3 %)	0.326
< Daily	428 (97.7 %)	206 (97.2 %)	222 (98.2 %)	reference
Heavy alcohol use (≥5 drinks per day)^a,c^				
Yes	13 (3.0 %)	4 (1.9 %)	9 (4.0 %)	0.263
No	425 (97.0 %)	208 (98.1 %)	217 (96.0 %)	reference
Engagement in sex work or drug dealing^a^				
Yes	164 (37.4 %)	72 (34.0 %)	92 (40.7 %)	0.145
No	270 (61.6 %)	138 (65.1 %)	132 (58.4 %)	reference
Incarceration^a^				
Yes	58 (13.2 %)	25 (11.8 %)	33 (14.6 %)	0.386
No	380 (86.8 %)	187 (88.2 %)	193 (85.4 %)	reference
HIV physician experience^b^				
Previously treated <6 patients	48 (11.0 %)	21 (9.9 %)	27 (11.9 %)	0.418
Previously treated ≥6 patients	340 (77.6 %)	170 (80.2 %)	170 (75.2 %)	reference
CD4 cell count^a^ (median, IQR)	300 (180–450)	360 (260–500)	230 (110–360)	<0.001
Years since HAART initiation^a^ (median, IQR)	5.7 (2.2–8.5)	5.9 (2.3–8.7)	5.5 (2.1–8.4)	0.243
Regimen^b^				
Single tablet per day	34 (7.8 %)	18 (8.5 %)	16 (7.1 %)	0.713
2-3 tablets per day	121 (27.6 %)	78 (36.8 %)	43 (19.0 %)	0.008
> 3 tablets per day	230 (52.5 %)	114 (53.8 %)	116 (51.3 %)	reference
Participation in inpatient addiction treatment^a^				
Yes	35 (8.0 %)	14 (6.6 %)	21 (9.3 %)	0.300
No	403 (92.0 %)	198 (93.4 %)	205 (90.7 %)	reference
Participation in outpatient detoxification or other outpatient addiction treatment^a,c^				
Yes	5 (1.1 %)	4 (1.9 %)	1 (0.4 %)	0.203
No	433 (98.9 %)	208 (98.1 %)	225 (99.6 %)	reference
Seeing drug counsellors or participation in addiction-related peer support meetings^a^				
Yes	43 (9.8 %)	30 (14.2 %)	13 (5.8 %)	0.003
No	395 (90.2 %)	182 (85.8 %)	213 (94.2 %)	reference
*Restricted to dual crack cocaine and opioid users (n = 293)*				
Participation in opioid substitution therapies^a^				
Yes	186 (63.5 %)	98 (72.6 %)	88 (55.7 %)	0.003
No	107 (36.5 %)	37 (29.4 %)	70 (45.4 %)	reference

*HAART* highly active antiretroviral therapy, *CI* confidence interval, *IQR* interquartile range, *pVL* plasma HIV RNA viral load

^a^ denotes activities/events in the past 6 months

^b^ denotes at the time of antiretroviral therapy initiation

^c^ p-values were derived from Fisher’s exact test

Not all cells added up to 100 % due to some missing values

Table 3 shows the results of univariable and multivariable GLMM logistic regression analyses of factors associated with optimal pharmacy refill adherence. As shown, in univariable analyses, older age (odds ratio [OR]: 2.29; 95 % confidence interval [CI]: 1.84 – 2.86), male gender (OR: 1.50; 95 % CI: 1.02 – 2.20), Caucasian ethnicity (OR: 1.61; 95 % CI: 1.12 – 2.32), higher CD4 count (OR: 1.39; 95 % CI: 1.30 – 1.48) and longer history of HAART (OR: 1.08; 95 % CI: 1.04 – 1.12) were significantly and positively associated with optimal pharmacy refill adherence. Homelessness (OR: 0.44; 95 % CI: 0.34 – 0.58), at least daily crack cocaine smoking (OR: 0.49; 95 % CI: 0.39 – 0.62), at least daily heroin use (OR: 0.32; 95 % CI: 0.22 – 0.47), and engagement in sex work or drug dealing (OR: 0.59; 95 % CI: 0.47 – 0.76) were significantly and negatively associated with optimal pharmacy refill adherence.

**Table 3:** Univariable and multivariable GLMM logistic regression analyses of factors associated with optimal pharmacy refill adherence for antiretroviral medications among HAART-exposed crack cocaine users in Vancouver, Canada, 2005–2013

Characteristic	Total sample of crack cocaine users (*n* = 438)				Dual crack cocaine and opioid users (*n* = 293)			
	Unadjusted OR (95 % CI)	*p*-value	Adjusted OR (95 % CI)	*p*-value	Unadjusted OR (95 % CI)	*p*-value	Adjusted OR (95 % CI)	*p*-value
Age^b^								
(per 10-year older)	2.29 (1.84 – 2.86)	<0.001	1.65 (1.33 – 2.04)	<0.001	2.75 (2.06 – 3.68)	<0.001	1.72 (1.30 – 2.26)	<0.001
Gender								
(male *vs.* female)	1.50 (1.02 – 2.20)	0.041			1.10 (0.68 – 1.79)	0.696		
Ethnicity								
(Caucasian *vs.* other)	1.61 (1.12 – 2.32)	0.011			1.40 (0.87 – 2.24)	0.167		
Education								
(<secondary *vs.* ≥secondary)	0.83 (0.57 – 1.20)	0.327			0.98 (0.61 – 1.58)	0.943		
Homeless^a,b^								
(yes *vs.* no)	0.44 (0.34 – 0.58)	<0.001	0.58 (0.44 – 0.77)	<0.001	0.39 (0.28 – 0.54)	<0.001	0.56 (0.40 – 0.79)	<0.001
Crack cocaine smoking^a,b^								
(≥daily *vs.* <daily)	0.49 (0.39 – 0.62)	<0.001	0.64 (0.50 – 0.81)	<0.001	0.41 (0.31 – 0.55)	<0.001	0.57 (0.43 – 0.75)	<0.001
Heroin use^a,b^								
(≥daily *vs.* <daily)	0.32 (0.22 – 0.47)	<0.001	0.43 (0.29 – 0.65)	<0.001	0.30 (0.20 – 0.46)	<0.001	0.45 (0.29 – 0.70)	<0.001
Crystal methamphetamine use^a,b^								
(≥daily *vs.* <daily)	1.07 (0.50 – 2.30)	0.858			0.79 (0.32 – 1.94)	0.609		
Heavy alcohol use^a,b^								
(≥5 drinks per day vs. <5 drinks per day)	1.17 (0.65 – 2.10)	0.596			1.37 (0.60 – 3.10)	0.452		
Engagement in sex work or drug dealing^a,b^								
(yes *vs.* no)	0.59 (0.47 – 0.76)	<0.001			0.54 (0.41 – 0.71)	<0.001		
Incarceration^a,b^								
(yes *vs.* no)	0.71 (0.49 – 1.02)	0.063			0.63 (0.41 – 0.96)	0.031		
HIV physician experience^c^								
(<6 patients *vs.* ≥6 patients)	0.74 (0.40 – 1.35)	0.322			0.60 (0.29 – 1.24)	0.169		
CD4 cell count^a,b^								
(per 100-cell increase)	1.39 (1.30 – 1.48)	<0.001	1.31 (1.23 – 1.41)	<0.001	1.43 (1.32 – 1.55)	<0.001	1.34 (1.23 – 1.45)	<0.001
Years since HAART initiation^a,b^								
(per year increase)	1.08 (1.04 – 1.12)	<0.001			1.11 (1.06 – 1.16)	<0.001		
Regimen^a,b^								
(1 pill per day *vs*. >3 pills per day)	1.23 (0.80 – 1.90)	0.339			1.29 (0.76 – 2.19)	0.347		
(2-3 pills per day *vs*. >3 pills per day)	1.01 (0.79 – 1.30)	0.920			1.07 (0.78 – 1.48)	0.659		
Participation in inpatient addiction treatment^a,b^								
(yes *vs.* no)	0.82 (0.57 – 1.18)	0.294			0.84 (0.54 – 1.32)	0.454		
Participation in outpatient detoxification or other outpatient addiction treatment^a,b^								
(yes *vs.* no)	0.65 (0.20 – 2.09)	0.472			0.43 (0.09 – 2.02)	0.284		
Seeing drug counsellors or participation in addiction-related peer support meetings^a,b^								
(yes *vs.* no)	1.06 (0.76 – 1.48)	0.730			0.84 (0.55 – 1.28)	0.424		
Participation in opioid substitution therapies^a,b^								
(yes *vs.* no)	–––		–––		1.71 (1.23 – 2.38)	0.001	1.39 (0.99 – 1.96)	0.056

*GLMM* generalized linear mixed-effect modelling, *HAART* highly active antiretroviral therapy, *OR* odds ratio, *CI* confidence interval

^a^ denotes activities/events in the past 6 months

^b^ denotes time-varying variables

^c^ denotes at the time of antiretroviral therapy initiation

In the final multivariable model, older age (adjusted odds ratio [AOR]: 1.65; 95 % CI: 1.33 – 2.04) and higher CD4 count (AOR: 1.31; 95 % CI: 1.23 – 1.41) remained independently and positively associated with optimal pharmacy refill adherence, while homelessness (AOR: 0.58; 95 % CI: 0.44 – 0.77), at least daily crack cocaine smoking (AOR: 0.64; 95 % CI: 0.50 – 0.81), and at least daily heroin use (AOR: 0.43; 95 % CI: 0.29 – 0.65) remained independently and negatively associated with optimal pharmacy refill adherence. When the sample was restricted to dual crack cocaine and opioid users, the same set of variables remained significantly associated with optimal pharmacy refill adherence. Participation in opioid substitution therapies was not independently associated with optimal pharmacy refill adherence (AOR: 1.39; 95 % CI: 0.99 – 1.96).

Table 4 presents the results univariable and multivariable GLMM logistic regression analyses of factors associated with pVL non-detectability. As shown, in addition to the covariates that were included for medication adherence, the final multivariable models included years since HAART initiation and antiretroviral tablet regimen. Longer history of HAART (AOR: 1.06; 95 % CI: 1.01 – 1.12) and receiving a single tablet-per-day regimen (AOR: 3.02; 95 % CI: 1.67 – 5.46) were independently and positively associated with pVL non-detectability. The results for the other covariates were consistent with those found in the multivariable model of medication adherence except that among dual crack cocaine and opioid users, participation in opioid substitution therapies was independently and positively associated with pVL non-detectability (AOR: 1.55; 95 % CI: 1.02 – 2.34).

**Table 4:** Univariable and multivariable GLMM logistic regression analyses of factors associated with pVL non-detectability among HAART-exposed crack cocaine users in Vancouver, Canada, 2005–2013

Characteristic	Total sample of crack cocaine users (*n* = 438)				Dual crack cocaine and opioid users (*n* = 293)			
	Unadjusted OR (95 % CI)	*p*-value	Adjusted OR (95 % CI)	*p*-value	Unadjusted OR (95 % CI)	*p*-value	Adjusted OR (95 % CI)	*p*-value
Age^b^								
(per 10-year older)	5.24 (3.72 – 7.37)	<0.001	1.86 (1.37 – 2.52)	<0.001	5.22 (3.45 – 7.89)	<0.001	1.59 (1.11 – 2.26)	0.011
Gender								
(male *vs.* female)	1.86 (1.10 – 3.15)	0.020			1.08 (0.59 – 1.97)	0.804		
Ethnicity								
(Caucasian *vs.* other)	1.31 (0.79 – 2.17)	0.290			1.38 (0.77 – 2.49)	0.282		
Education								
(<secondary *vs.* ≥secondary)	1.03 (0.62 – 1.72)	0.909			1.39 (0.77 – 2.49)	0.272		
Homeless^a,b^								
(yes *vs.* no)	0.26 (0.19 – 0.36)	<0.001	0.39 (0.28 – 0.54)	<0.001	0.24 (0.17 – 0.35)	<0.001	0.37 (0.25 – 0.56)	<0.001
Crack cocaine smoking^a,b^								
(≥daily *vs.* <daily)	0.47 (0.36 – 0.61)	<0.001			0.39 (0.29 – 0.53)	<0.001	0.69 (0.49 – 0.96)	0.028
Heroin use^a,b^								
(≥daily *vs.* <daily)	0.35 (0.23 – 0.54)	<0.001	0.60 (0.36 – 0.98)	0.042	0.32 (0.20 – 0.51)	<0.001		
Crystal methamphetamine use^a,b^								
(≥daily *vs.* <daily)	1.09 (0.45 – 2.62)	0.853			1.15 (0.43 – 3.10)	0.779		
Heavy alcohol use^a,b^								
(≥5 drinks per day vs. <5 drinks per day)	0.70 (0.36 – 1.37)	0.302			0.57 (0.24 – 1.37)	0.209		
Engagement in sex work or drug dealing^a,b^								
(yes *vs.* no)	0.58 (0.44 – 0.75)	<0.001			0.58 (0.43 – 0.78)	<0.001		
Incarceration^a,b^								
(yes *vs.* no)	0.57 (0.38 – 0.86)	0.008			0.49 (0.31 – 0.78)	0.002		
HIV physician experience^c^								
(<6 patients *vs.* ≥6 patients)	1.15 (0.51 – 2.62)	0.738			0.84 (0.35 – 2.00)	0.688		
CD4 cell count^a,b^								
(per 100-cell increase)	1.93 (1.76 – 2.13)	<0.001	1.67 (1.52 – 1.83)	<0.001	1.98 (1.77 – 2.21)	<0.001	1.72 (1.54 – 1.91)	<0.001
Years since HAART initiation^a,b^								
(per year increase)	1.27 (1.21 – 1.34)	<0.001	1.06 (1.01 – 1.12)	0.012	1.29 (1.22 – 1.37)	<0.001	1.08 (1.02 – 1.14)	0.011
Regimen^a,b^								
(1 pill per day *vs*. >3 pills per day)	3.11 (1.76 – 5.52)	<0.001	3.02 (1.67 – 5.46)	<0.001	2.84 (1.45 – 5.57)	0.003	2.60 (1.30 – 5.21)	0.007
(2-3 pills per day *vs*. >3 pills per day)	1.70 (1.27 – 2.30)	<0.001	1.24 (0.91 – 1.69)	0.179	1.60 (1.13 – 2.29)	0.009	1.14 (0.79 – 1.65)	0.493
Participation in inpatient addiction treatment^a,b^								
(yes *vs.* no)	0.67 (0.45 – 1.02)	0.061			0.58 (0.35 – 0.97)	0.037		
Participation in outpatient detoxification or other outpatient addiction treatment^a,b^								
(yes *vs.* no)	1.10 (0.33 – 3.71)	0.872			1.63 (0.34 – 7.74)	0.539		
Seeing drug counsellors or participation in addiction-related peer support meetings^a,b^								
(yes *vs.* no)	1.32 (0.90 – 1.94)	0.158			1.16 (0.73 – 1.84)	0.532		
Participation in opioid substitution therapies^a,b^								
(yes *vs*. no)	–––		–––		2.28 (1.58 – 3.30)	<0.001	1.55 (1.02 – 2.34)	0.039

*GLMM* generalized linear mixed-effect modelling, *pVL* plasma HIV RNA viral load, *HAART* highly active antiretroviral therapy, *OR* odds ratio, *CI* confidence interval

^a^ denotes activities/events in the past 6 months

^b^ denotes time-varying variables

^c^ denotes at the time of antiretroviral therapy initiation

## Discussion

Among our sample of HAART-exposed crack cocaine users, we found that homelessness, and high-intensity crack cocaine and heroin use were independently and negatively associated with optimal pharmacy refill adherence and pVL non-detectability. Except for opioid substitution therapies, none of the addiction treatment modalities assessed, including inpatient treatment, outpatient detoxification services, drug counselling, and participation in peer support groups such as Narcotics Anonymous, were significantly associated with optimal pharmacy refill adherence or pVL non-detectability. Specifically among a sub-sample of dual crack cocaine and opioid users, enrolment in opioid substitution therapies was independently associated with pVL non-detectability but not with optimal pharmacy refill adherence.

We found an independent association between at least daily use of crack cocaine and poor medication adherence and having a detectable viral load. This is consistent with previous reports that crack cocaine users are less likely to enjoy gains from HAART [13–15]. In addition, the finding that at least daily use of heroin was also independently associated with suboptimal pharmacy refill adherence and outcome indicates a need to address poly-substance use among HIV-infected crack cocaine users. Previous research suggested that poly-substance use is quite common among people who use drugs, although those who primarily use cocaine may continue to use it consistently over an extended period of time while keeping the use of other non-primary drugs at relatively lower levels [23]. While current literature suggests no clinically significant pharmacokinetic interactions between crack cocaine and antiretroviral agents, methamphetamines may have possible lethal interactions with antiretroviral agents that inhibit the CYP450 system [24]. In our sample of HIV-positive crack cocaine users, high-intensity use of crystal methamphetamine was not common or associated with optimal pharmacy refill adherence or pVL non-detectability; however, in a setting where co-use of crack cocaine and crystal methamphetamine is common, a greater caution is required when prescribing HAART. Collectively, these findings emphasize the need to ensure that a variety of addiction treatment options be integrated with HIV treatment and care for individuals who use crack cocaine.

The finding that none of the addiction treatment modalities except for opioid substitution therapies were associated with optimal pharmacy refill adherence or viral suppression serves to underscore previous calls for identifying more evidence-based addiction treatment approaches that also confer benefits on HIV disease management among HIV-positive crack cocaine users [5, 14, 25]. While a previous pilot study suggested that a video-based behavioural intervention employing the Information-Motivation-Behavioural Skill model might be efficacious in reducing crack cocaine use and improving medication adherence among HIV-positive crack cocaine users [26], the efficacy of the intervention needs to be determined with a larger sample. Also, the long-term post-intervention effect on crack cocaine use and medication adherence is an important outcome to be examined, as a Cochrane Collaboration systematic review has reported that many behavioural interventions for substance use suffer from a lack of the sustained effect following the intervention [27]. Further, unlike the case of opiate addiction, there is currently no approved medication to treat crack cocaine addiction. While many candidate medications have been evaluated for the efficacy in the treatment of crack cocaine/cocaine addiction during the past decade, overall results have been inconclusive [28–30]. Further research is needed in this area.

In contrast, we found that dual crack cocaine and opioid users who were enrolled in opioid substitution therapies were more likely to achieve virological success, and the positive effect of opioid substitution therapies remained significant even after the adjustment for the high-intensity use of crack cocaine and heroin. This adds important evidence to a body of research indicating the effectiveness of these therapies for the improvement of HIV treatment adherence and outcomes [31]. Previous studies reported that ongoing cocaine use undermined the effectiveness of opioid substitution therapies for HIV treatment-related outcomes among HIV-infected patients accessing these therapies [32, 33]. However, our findings suggest that opioid substitution therapies still help HIV-infected dual crack cocaine and opioid users achieve optimal HAART outcomes. Nonetheless, as crack cocaine/cocaine users have been shown to have significant barriers to adherence and continuance of opioid substitution therapies [34, 35], adjunct interventions to opioid substitution therapies that help retain crack cocaine/cocaine-using patients in treatment need to be developed.

Similarly to our previous investigation that indicated that homelessness posed a barrier to antiretroviral therapy among HIV-infected people who use illicit drugs generally [36], we found that homelessness was independently associated with suboptimal pharmacy refill adherence and viral suppression failures among HIV-infected individuals who use crack cocaine in a setting where HAART is provided free of charge. Collectively, these findings indicate that, in addition to identification of evidence-based addiction treatment strategies for crack cocaine use, structural interventions to address homelessness in general (e.g., housing assistance) are needed. Expanding and promoting support services for HIV-positive homeless individuals focusing on both access and adherence to HAART would maximise the benefits of HAART on disease progression and onward viral transmission in this population.

Our study has several limitations. First, as the study sample was not randomly selected, our findings may not be generalizable to other populations of crack cocaine users. Second, the self-reported data may be affected by reporting biases, including recall bias and socially desirable responding. However, we note that this type of data has been commonly utilized in observational studies involving people who use drugs and found to be valid [37]. Also, as our primary outcomes were ascertained through a confidential record linkage to the provincial HIV-related clinical database, we believe that it is unlikely that potential reporting biases have impacted the data differentially by the medication adherence or pVL levels. Third, as with all observational studies, the relationships between the explanatory variables and outcome assessed may be under the influence of unobserved confounding, although we sought to address this bias with multivariable adjustment of the key demographic, behavioural, and clinical predictors of medication adherence or viral suppression.

## Conclusions

In sum, among our sample of HAART-exposed crack cocaine users, those who were homeless and those who engaged in high-intensity crack cocaine or heroin use were more likely to have suboptimal adherence to antiretroviral medications and fail to achieve viral suppression. Further, none of the addiction treatment modalities assessed appeared to facilitate medication adherence or viral suppression, except for opioid substitution therapies. These findings suggest an urgent need to develop evidence-based treatment options for crack cocaine use that also confer benefits to HAART-related outcomes.

## Abbreviations

ACCESS, the AIDS Care Cohort to evaluate Exposure to Survival Services; AIC, Akaike information criterion; AOR, adjusted odds ratio; GLMM, generalized linear mixed-effects modelling; HAART, Highly-active antiretroviral therapy; IQR, interquartile range; OR, odds ratio; pVL, plasma HIV RNA viral load; TasP, Treatment as Prevention
